# Nanotechnology in Modern Photodynamic Therapy of Cancer: A Review of Cellular Resistance Patterns Affecting the Therapeutic Response

**DOI:** 10.3390/pharmaceutics12070632

**Published:** 2020-07-06

**Authors:** Elvin Peter Chizenga, Heidi Abrahamse

**Affiliations:** Laser Research Centre, Faculty of Health Sciences, University of Johannesburg, Johannesburg 2028, South Africa; elvinc@uj.ac.za

**Keywords:** photodynamic therapy (PDT), photosensitizer (PS), cellular resistance, nanoparticles (NPs), drug delivery systems (DDS), pharmacokinetics

## Abstract

Photodynamic therapy (PDT) has emerged as a potential therapeutic option for most localized cancers. Its high measure of specificity and minimal risk of side effects compared to other therapies has put PDT on the forefront of cancer research in the current era. The primary cause of treatment failure and high mortality rates is the occurrence of cancer resistance to therapy. Hence, PDT is designed to be selective and tumor-specific. However, because of complex biological characteristics and cell signaling, cancer cells have shown a propensity to acquire cellular resistance to PDT by modulating the photosensitization process or its products. Fortunately, nanotechnology has provided many answers in biomedical and clinical applications, and modern PDT now employs the use of nanomaterials to enhance its efficacy and mitigate the effects of acquired resistance. This review, therefore, sought to scrutinize the mechanisms of cellular resistance that affect the therapeutic response with an emphasis on the use of nanomaterials as a way of overriding cancer cell resistance. The resistance mechanisms that have been reported are complex and photosensitizer (PS)-specific. We conclude that altering the structure of PSs using nanotechnology is an ideal paradigm for enhancing PDT efficacy in the presence of cellular resistance.

## 1. Introduction

The rapid effort in the search for new cancer therapies has in recent years, made a significant impact in cancer and biomedical research. At present, numerous therapeutic options, including hormone therapies, gene expression modulators, immunotherapies, apoptosis inducers, angiogenesis inhibitors, hormone therapies, signal transduction inhibitors, therapeutic vaccines, and gene therapy, have been employed for treating different cancers, which have shown improved cancer therapy and prognosis [1,2,3]. Another benefit from the field of cancer research is the advent of therapies with an interdisciplinary approach involving a close-fitting association between complex processes in biology, biophysics, and biochemistry, which ultimately aim at achieving targeted tumor eradication. The complexity in such therapies is a very useful feature for cancer therapies since it provides a solution to most of the hurdles in treating tumors. Due to their altered cell signaling, cancer cells not only grow rapidly, but also have enhanced survival dispositions [4] that, in turn, make putative therapies ineffective and rather lethal to normal tissue.

Photodynamic therapy (PDT) is one example where a complex interplay between all these scientific domains is applied. It employs the use of two individually distinct elements, i.e., a photoactivatable drug called a photosensitizer (PS) and light, especially from lasers, to achieve one purpose [5]. This feature gives PDT a high measure of specificity and minimal risk of side effects when compared to other therapies and, hence, PDT has been on the forefront of cancer research in the current era. As an anticancer therapy, PDT kills cancer cells through oxidative stress produced by the highly cytotoxic Reactive Oxygen Species (ROS), generated by the PS in its activated state. The molecular mechanisms involved in the PDT process have been amply elucidated and characterized in literature [6]. The main form of ROS produced in PDT is singlet oxygen (^1^O_2_), which initiates reactions and leads to activation of apoptosis, necrosis, and macro-autophagy (MA) in cells as well as activation of the immune system and the destruction of tumor vasculature in vivo [7,8]. The establishment of PDT as an alternative treatment modality for most localized cancers has given more hope for the possibility of maximum cancer eradication with a good prognosis of cancer. Although most studies are still in vitro and some in clinical trials, PDT has currently been approved for treating topical lesions and several types of cancers including, but not limited to, cancer of the esophagus, papillary bladder, lung, and melanoma [9]. PDT has many advantages over other therapeutic options and studies have shown and proven that PDT is a preferred therapeutic option for many cancers [10]. 

Nevertheless, major concerns of cancer cell resistance to PDT have emerged despite its thorough thought-out approach. There are instances where PDT can be rendered ineffective or, in extreme cases, trigger lethal therapeutic outcomes including cancer propagation, if administered incorrectly [11]. In the early days of PDT studies, numerous issues that brought challenges in the use of PDT have been addressed and corrected over the years. Such issues include PS hydrophobicity, which has been corrected by metallizing hydrophobic PSs to render them more water soluble [12,13]. Another setback was the issue of PS dimerization, which has been corrected by increasing the zeta potential, which stabilizes PSs through steric repulsion [14]. Activation wavelength was also once a limiting factor in the use of PDT for deeper tissues, but now the increased use of PSs that are activated in the red region (therapeutic window), the wavelength at which tissues are idyllically transparent, has solved this barrier. However, the matters arising from recent PDT research show increasing levels of PDT resistance. Despite its numerous advantages over most therapies, the resistance patterns in PDT are possible causes of treatment failure as is the case with chemotherapy.

Nanotechnology has, thus far, provided many answers to the problems of PDT and its applications. Especially due to the contemporary reports of cancer cell resistance to PDT, in recent years [15], the manipulation of matter on nanoscale sizes is of paramount importance in the use of PDT. The resistance patterns and mechanisms involved in PDT have been described and this has led to the development of efficient PS modification mechanisms, e.g., functionalization and conjugation to nanoparticles (NPs) and immune agents, and the advent of other drug delivery systems (DDS), e.g., liposomes and nanotubes [10,16]. This review, therefore, sought to examine the cellular resistance patterns in PDT and the use of nanotechnology to discourse at great length on the biochemical and biological interplays that affect the therapeutic responses.

## 2. Photodynamic Therapy

### 2.1. Basic Pharmacokinetic and Pharmacodynamics of PDT

**Figure 1:**
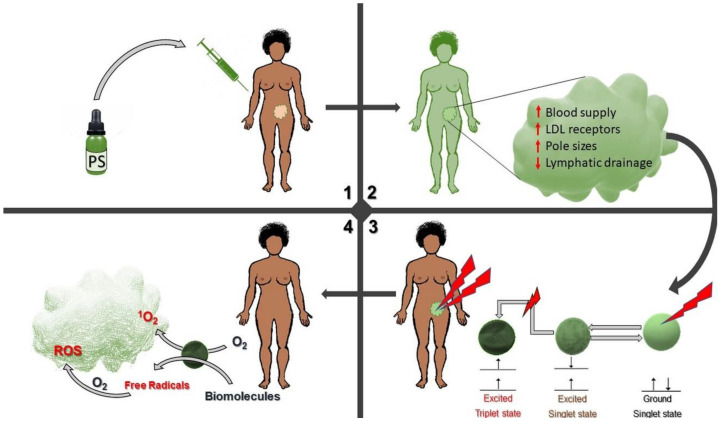
Schematic representation of photodynamic therapy (PDT) of cancer showing the administration of photosensitizer (PS), its actions, and the photodynamic process to achieve a tumor. 1. The PS is administered into the body orally or intravenously. 2. The PS circulates in the vascular system and selectively accumulates in the tumor cells. 3. Light is specifically directed to the tumor region and activates the PS 4. The PS in its activated state causes cytotoxic reactions to repress the tumor.

For any therapeutic option, the main themes to evaluate are the pharmacokinetic and pharmacodynamic factors, which are utterly vital in determining the therapeutic outcome. The distinctiveness of PDT with other therapies lies in its high selectivity for tumor cells. The two nontoxic components, the PS and light, can produce cytotoxicity after a physical interaction at the site of the tumor. The PS selectively localizes in tumors and tumor vasculature due to many characteristics of the tumor (Figure 1) and stays inside the cancer cells until light is irradiated onto the tumor. The activating light is then focused onto the tumor area to activate the absorbed PS detonating an added level of selectivity where the rest of the body tissues are not affected. The light is of an appropriate wavelength and energy, usually laser, although other light sources, e.g., polychromatic light can be used for other cancers, especially those affecting the superficial layers of the body [17]. Though the PS and light are the essential components in PDT, there is a third component necessary for PDT to occur, i.e., the inherent molecular oxygen present in the tissue extracellular and intracellular spaces, which is readily available to be a substrate for the activated PS to form ROS. Depending on the cancer, the PS can be administered locally or systemically via injections. At the PS administration stage, there is a high level of tumor selectivity due to various factors including the high tumor vasculature that increases the surface area for PS entry in cancer tissue than normal tissue; The increased membrane permeability of cancer cells other than normal cells due to overexpression of low-density lipoprotein (LDL) membrane receptors; and also the decreased lymphatic drainage of tumor tissue [18]. All these characteristics of cancer tissue increase their affinity for PS entry alongside the specific and focused application of light only at the tumor site (Figure 1). 

Eventually, after irradiation, either of three mechanisms achieve the therapeutic outcome. These also depend on the PS site of localization. Most prominent is direct tumor cell death by apoptosis or necrosis, which occurs when the PS was taken up by tumor cells and localized in either one or more of the different cellular organelles including cell membrane, mitochondria, lysosomes, and endoplasmic reticulum [8]. At the point where the PS accumulates in the cells, there are two classes of reactions that can occur simultaneously: photooxidation by free radicals (type I reaction) and photooxidation by ^1^O_2_ (type II reaction) [6,8]. Although these reactions occur simultaneously, one can hypothesize that the type of reaction preferred would depend on the tumor microenvironment. For instance, the availability of oxygen in the cancer tissue would favor type II reaction and, in cancers that are highly hypoxic, type I reaction would be more prominent. Some studies have also shown that the balance between type I and type II reactions depend on the PS being used, affinity of the PS with the substrate, and the concentrations of oxygen and substrate [19,20]. Ding et al. [21] studied the photoactivation switch from Type II to Type I reactions in hypoxic tumor cells by using micelles and noted that these carriers modulated the resulting reaction under different microenvironments.

In the second mechanism, the PS that adsorbed to the dense blood vessel network of tumor tissues causes potent anti-vascular effects that destroy the tumor vasculature leading to thrombosis and hemorrhaging that subsequently, lead to tumor death via deprivation of oxygen and nutrients. Lastly, an ancillary mechanism that follows is the direct activation of the immune system [22]. When the tumor cells and vasculature are stressed due to the PDT effect, acute inflammation is induced, and it is heightened by the release of cytokines and stress response proteins from the dying tissue. This leads to an influx of leukocytes that can both contribute to tumor destruction as well as stimulation of the immune system to recognize and destroy tumor cells [23]. 

The one thing with which every novel therapeutic option is trying to achieve is the selective eradication of tumor cells without harming healthy cells. In tissues that have the potential to regenerate post cancer treatment, preservation of the normal cells during therapy would allow for total recovery without frailty. The mechanism through which PDT works provides this desired therapeutic outcome unlike most therapies, such as chemotherapy and radiation. However, the emergence of cell resistance to PDT as described below also stretches out an area of major concern.

### 2.2. Mechanisms of Resistance in PDT

The products of type I and type II reactions of PDT, i.e., ROS and ^1^O_2_, respectively, are chemically very reactive molecules and have a very short half-life, which results in specific parts of the cells that are within a 20-nm radius being affected [24]. This minimizes the therapeutic effect on normal cells. Because of this short life span of PDT products, PS localization is a primary factor with great importance in the success of PDT. Different PSs have an affinity for different parts of the cell and, hence, the PSs can localize in different organelles. Regardless of where the PS localizes, cytotoxicity occurs through disruption of membrane and intracellular proteins that subsequently leads to activation of apoptosis, autophagy, and/or necrosis. 

Apart from the direct cellular cytotoxicity of PDT, numerous studies have reported on other significant effects of PDT on gene expression and cell signaling. Several studies show that the PDT process induces activation of different cell signal transduction pathways and the expression of other extracellular signal regulated kinases [25,26,27,28,29]. PDT also results in activation of antiapoptotic Bcl-2 proteins [30] and stimulation of the autophagic response of cells [31,32]. Castano and colleagues [33] reported on the important aspects involved in the chemical structure, photochemistry, photo-physics, and subcellular localization of PSs, and the changes in cellular metabolism and intracellular signaling and modes of cell death in PDT [34]. In the end, the complex reactions of cells from the interaction with PDT products provides a platform through which cancer cells acquire resistance to PDT.

Recently, cancer resistance to therapy is a recognized paradigm and a major concern in clinical oncology. Although resistance to most chemotherapeutic drugs may be attributed to a range of genetic variations and individual differences, the cancer cell’s ability to resist drugs is ascribed to several common intrinsic properties of all cancer cells. Enough evidence also shows that another cause of treatment resistance is the heterogeneous nature of tumors with cancer stem cells (CSCs) at the core of the tumor [35,36,37,38]. CSCs are responsible for many processes including tumorigenesis, tumor maintenance, metastasis, treatment resistance, and post-treatment tumor recurrence [39,40]. Their mechanism of resistance is attributed to many inherent properties including the presence of ATP-Binding Cassette (ABC) transporters, slow cell kinetics, stem cell signaling pathways, overexpression of DNA repair proteins, and their existence in hypoxic niches. It is not surprising that, in PDT, cancer cell resistance has also been noted and reported. As seen in chemotherapy, drug efflux is also a cause of resistance in PDT, where the PSs are pumped out before their action [41,42,43]. Numerous drug efflux proteins associated with chemotherapy resistance have also been implicated in PDT resistance including multidrug resistance (MDR) phenotype [41], P-glycoprotein (P-gp) [15], and ATP-binding cassette super-family G member 2 (ABCG2) [15,43,44]. 

However, though not widely discussed in the episteme of PDT, there have emerged other numerous intracellular mechanisms by which cells resist PDT. As shown in Table 1 below, these mechanisms have been amply characterized and reported in common cancer cell lines. A better understanding of these mechanisms, therefore, is necessary for improving PDT as an option of cancer therapy. With second and third generation PSs, the predicament of getting the PS internalized by the cancer cells was taken care of since these PSs are more hydrophilic and, with the use of metalized PS molecules, PS uptake is assured. However, most of these resistance mechanisms are acquired through biological events that occur during and after cell-drug (PS) interactions, which occur during the photodynamic process. Common resistance patterns are summarized in Table 1 below.

Earlier studies by other groups [46,52] confirm that treatment of cancer cells alters the expression of many cellular functions including the levels of stress responsive proteins, known as the Heat Shock Proteins (HSPs). HSPs are found in all major cellular compartments and play an important role in thermal stress and protein homeostasis during stressful conditions [53]. Although ironically some HSPs influence anti-cancer properties, as is the case of curcumin in colon cancer cells reported by Liang et al. [54], they commonly assist in cell survival and resistance to apoptosis through repair and refolding of misfolded and damaged peptides [55,56]. In PDT, HSP-mediated inhibition of apoptosis has been reported in previous studies [57]. This implies a possible resistance mechanism to putative PDT. Furthermore, in as much as ROS, are products of PDT responsible for cell cytotoxicity, biologically, they are also common by-products of normal metabolic processes, which serve as essential signaling mediators in vital processes including cellular proliferation, aging, physiologic death, and many other cellular processes [58]. As described previously, cells may follow either apoptotic signaling, autophagy, or other cell fate mechanisms depending on various physiological changes intercellularly and intracellularly. Autophagy is one of the cell death pathways post-PDT. However, macro-autophagy specifically contributes to acquisition of some resistance patterns. Macro-autophagy is designed to remove damaged or unnecessary organelles, but the subcellular site of ROS production, the type of ROS, and the modified targets, are very crucial factors that determine the pro-death or pro-survival functions of macro-autophagy. Dewaele et al. [32] found that, besides the increased apoptosis in PDT-treated cells, attenuation of macro-autophagy enhances the accumulation of ROS-damaged proteins, which leads to the activation of cell pathways that remove ROS damaged cytoplasmic components. Hence, this prevents damage by ROS generated during PDT.

Ji et al. [59] found another intriguing phenomenon in PDT-treated human esophageal normal Het-1A cells, which had a high expression of Hypoxia-Inducible Factor1 (HIF1)-alpha. Their findings suggested that PDT-induced tissue hypoxia as a result of vascular damage and oxygen consumption would limit the efficacy of PDT. Colleagues elsewhere have also found and described other mechanisms of PDT resistance including, but not limited to, the changes in mitochondrial size and function, alterations in the enzymatic pathways, delays in apoptotic responses, and many more (Table 1). To date, many resistance mechanisms have been reported in different tumors and cell lines. Additionally, there is a strong association of resistance to the individual PSs and not the photosensitization itself and, hence, different cancer cells have been recognized for their resistance to different types of PSs by varying mechanisms. Mayhew et al. [60] evidently presented that the mechanism of PDT resistance may depend upon the physical nature of individual PSs. Additionally, the conflicting results regarding co-resistance between PDT and other putative therapies further explain the existence of a wide array of varying resistance mechanisms. In the end, PDT resistance poses a major challenge that needs proper interventions.

## 3. Nanotechnology in PDT

To maximize cancer eradication amid the trends in cell resistance to therapy and the indeterminate PS delivery, modern research in cancer has seen a rampant increase in the investigation of nanomaterials and their use in biological and medicinal applications. PDT has also scooped a measurable amount of benefits from these recent advances in nanotechnology. Due to the complexity of the pharmacological and immunological interactions involved in PDT, and the numerous reports on PDT resistance, ideas to modify the PS, its potency, and how it is delivered to the target tissue using nanotechnology, have emerged and stand out to be very effective options for PDT. Additionally, though most nanomaterials are useful only as carrier molecules, some have in themselves, shown photoactive properties that can be explored for their use in PDT.

The goal of any cancer therapeutic is to destroy the tumor cells while minimizing damage to normal cells. In resistant tumors, increasing the dose of the PS or the irradiation time should not be an option. We demonstrated in a study [11] that high PS concentrations and long exposure times result in the uptake of the PS by normal cells, which, subsequently, would cause an unfavorable PDT outcome and possibly photosensitivity during and after treatment. Moreover, the structure of the PS is the main factor in acquisition of resistance [15], and because the cytotoxic species have a short life span and can only affect substrates within a 20-nm radius [24], where the PS localizes in the tumor, and intracellular PS concentration, are very important in the final effect of PDT. For such reasons, altering the structure of the PS is the ideal approach for mitigating the problem of cellular resistance. Moreover, another cause of substandard PDT outcome is the problem of drug delivery to the target tissue, and nonspecific distribution of the PS in the body, which limit the drug potency with a concomitant increase of side effects. 

To maximize the efficacy of PDT, therefore, targeted delivery of the PS using nanotechnology is paramount. Numerous nanomaterials have been studied for use in DDS. Ideally, the choice of nanomaterial used depends on many factors including, but limited to, the desired therapeutic effect, type of PS, the preferred target, cost, and stability (Table 2). 

When used in DDS, nanomaterials can (1) protect the PS against enzymatic degradation, (2) control PS release allowing a constant and uniform concentration into target cells, (3) facilitate entry of PS into the target cells, (4) can be conjugated to multiple drug molecules that increases drug load and also simultaneously enable combinatory cancer therapy, and (5) they can bypass common drug resistance mechanisms [16,73,74]. Nanomaterials have been extensively studied for their use in different applications including DDS for chemotherapy and emerging PDT, molecular and cellular imaging, biomarker detection, and several others. Apart from a few pharmacokinetics flouts, no resistance to nano-based DDS has been reported in literature to this day. 

In the presence of many different types of nanomaterials used as shown in Table 2 above, dendrimers, liposomes, and metal NPs are researched more for PDT drug delivery. The wide range of nanomaterials currently available for use in cancer treatment possess many features that can be explored for applicability in the attenuation of the resistance patterns previously described (Table 1). Notably, though most resistant patterns are positioned toward the PS, a couple of mechanisms have a common interplay with chemotherapy resistance. Correspondingly, most of these nanomaterials have shown enhanced potency and reduced resistance in both chemotherapy and PDT. In all practical senses, though some features have been noted as effective for chemotherapy, a similar approach can be inferred to PDT and the attenuation of PDT resistance.

### 3.1. Attenuating Cellular Resistance Using Nanotechnology

As discussed earlier, increasing the dose of PDT would not resolve the problem of resistance cordially. Most nanomaterials, therefore, provide a solution for attenuation of resistance by increasing drug load to individual cells through active targeting and increased uptake. Some possess properties to override specific resistance mechanisms, some can disrupt cell repair systems, and some can directly enhance the photosensitization process. In specific instances, the mechanisms of attenuation have been amply described and, hence, provided more knowledge that can be used to design better photosensitization models. With reference to Table 1, the section below presents some of the nanotechnological approaches suitable for attenuation of cellular resistance to PDT.

#### 3.1.1. Modulation of the PS Uptake and/or Subcellular Localization

Certain cancer cells evade treatment by preventing the entry of the PS or altering the localization of the PS inside the cell [15,75]. This mechanism results in the unavailability of the PS, which results in unsuccessful cell death when light is introduced. Hence, receptor targeting by using molecules that bind membrane receptors on cell surfaces or those that target membranes of intracellular organelles is a very significant solution to the problem of impaired PS update and its intracellular kinetics. Receptor targeting molecules can be conjugated to NPs in properly designed DDS systems to achieve this purpose. Therefore, the concept of DDS being widely and routinely used in many therapeutics, is also an essential component in attenuating cellular resistance. In addition to that, certain NPs have the propensity to enter cells by electrostatic attraction and phagocytic internalization [76]. Once inside the cells, they are able to freely localize in the cytoplasm and affect cellular organelles.

Our group [77] and others [78,79,80] have extensively studied the effect of nano-based PDT to enhance PS uptake. A multi-component compound engineered using metallated phthalocyanine, poly ethylene glycol (PEG), a gold (Au) NP, and an antibody against the melanoma inhibitory activity, an antigen highly expressed on melanoma cells, was used for treating melanoma cells and showed enhanced PDT in vitro [77]. Such a compound possesses many functions including specific targeting, binding, and increased cellular uptake of the PS. By doing so, cellular resistance acquired by modulation of PS uptake in cells that decrease the expression and function of LDL receptor, involved in the transport of certain PSs, can be avoided. Similarly, in another study, a multicomponent drug conjugate comprising of an antibody against the breast cancer specific antigen, the human epidermal growth factor receptor 2 (HER2), zinc phthalocyanine, and PEGylated AuNP was synthesized for treating breast cancer cells [80]. The compound indicated stability toward aggregation, and, when used in PDT, showed efficient production of cytotoxic ^1^O_2_. In addition to the marked cytotoxicity indicated, the compound demonstrated selective targeting of breast cancer cells that overexpress the HER2, via immunomodulatory interaction between the drug and the cells. Matsuzaki et al. [81] studied the effects of an anti-glypican-1 antibody-drug conjugate comprised of a cytotoxic drug monomethyl auristatin F (MMAF) and an antibody against Glypican-1 (GPC1), which is highly expressed in solid tumors. They presented that the drug conjugate showed enhanced uptake and antitumor activity in glypican-1 positive uterine cervical cancer cells.

PDT has also benefited from the exciting field of research that employs the use of aptamers due to their specific targeting ability and enhanced membrane transfer. These molecules are small single-stranded Deoxyribonucleic Acid (DNA) or Ribonucleic Acid (RNA) oligonucleotides that can bind target molecules with high affinity and specificity, which is analogous to the action of antibodies [82]. Historically, one can credit the discovery of aptamers to the emergence of the Human Immunodeficiency Virus (HIV) in the early 1980s. During the time when HIV was developing to become a public health problem, research on HIV in search of therapies and management strategies led to the discovery of fascinating RNA transcripts that bound to viral or cellular proteins with high affinity and specificity [83]. These molecules were encoded by the viral particle to modulate the activity of proteins essential for their replication or to inhibit the activity of proteins involved in cellular antiviral responses. It was after that time when scientists hypothesized that these molecules can be synthesized to specifically target proteins in the body for diagnostic and therapeutic purposes. 

Since the discovery of aptamers 40 years ago, a few research groups have investigated their application in PDT and DDS. Aptamers are small with most of them ranging from 20 to 60 nucleotides long and have a higher tissue absorption rate. Their small size allows them to be used for both surface biomarker recognition and intracellular targeting. In previous studies, aptamer-NP conjugates have certainly shown better tissue penetration [84,85]. Recently, Kim et al. [85] developed a tumor-specific aptamer-conjugated polymeric PS for treating gastrointestinal cancer using an AS1411 aptamer, which binds to nucleolin on the membrane of cancer cells. This compound was use in a laparoscopy-based PDT and their results showed enhanced and effective eradication of cancer cells under laser irradiation. Similarly, another group [86] previously studied a similar molecule that showed tight binding between the PS and the aptamer by intercalation and outside binding. When used to treat MCF7 cells, there was marked photodamage compared to cells that did not express nucleolin. These molecules are, therefore, potential candidates for specific targeting, nuclear targeting, and enhancement of PS uptake in cells that modulated PS uptake and localization. Aptamers can be conjugated to an array of PSs using a NP core to target cellular components for enhanced uptake and directed localization. Even with intracellular targets as desired recognition molecules, aptamers can be used. Overriding the modulation of PS uptake and localization by resistant cells can therefore benefit from the use of aptamers due to their immense specific targeting potential, and ease of modification. 

Using such innovations, no observations have been reported on continued resistance, this far. This is a result of the specific binding, which increases the amount of time the PS is available for active transportation into the cells, even in the presence of reduced carrier molecules. Inside the cell, alteration of PS kinetics to cellular organelles is also avoided when intracellular targets, including the nucleus, mitochondria, and other cytoplasmic organelles, are directed. The added advantages with the use of these molecules include their low cost, non-immunogenic nature and the fact that they can be developed for a wide range of cellular targets. 

#### 3.1.2. Enhanced Damage Repair and Evasion of Apoptosis

Other cell types do not confer resistance by avoiding PS entry, but rather using mechanisms that prevent damage after the photosensitization. In these cases, the PS and light would both be effective in function, which causes notable photosensitization in the cell. However, due to acquired mechanisms, these resistant subtypes prevent the induced damage. In one study, it was observed that ALA-PDT resistant subtypes had a higher concentration of intracellular proteins and increased number of mitochondria [49]. To combat the effect of enhanced damage repair and evasion of apoptosis, details of which have been discussed in preceding sections (Section 2.2 above), nanomaterials can be used to increase intracellular drug load and concentration, which, in turn, increases the amount of reactions produced and, hence, exceeds the rate at which the cell is able to repair the induced damage. Thus, the more the PS concentration is, the higher the PDT effect is and, consequently, the less likely a resistant cell is able to repair damage. This intervention is applicable in cells that use enhanced DNA repair mechanisms, increased HSPs, Increased HIFs, and other repair molecules.

Consequently, certain NPs not only attenuate the effects of increased damage repair and evasion of apoptosis by increasing drug load, but also affect cellular pathways directly. Some NPs, especially metal NPs, directly modulate autophagy and the apoptotic pathways. As mentioned previously, macro-autophagy confers cellular resistance by aiding in the removal of ROS-damaged cytoplasmic components post-treatment. However, modulation of the autophagy responses that result in more cellular damage and activation of apoptosis is a desired effect of therapies including PDT. Recent studies show that certain nanomaterials like iron-based NPs (FeNPs) possess direct cytotoxic effects by inducing oxidative stress and, especially when combined with other factors, their effects can alter intracellular signaling, which directly contributes to apoptosis [87]. The latter can be achieved in instances where an appropriate PS is conjugated to these FeNPs to treat resistant cells. The combined effect of PDT induces ROS and ^1^O_2_ with the oxidative stress induced by FeNPs that can potentially evade the cells’ ability to repair damaged molecules before apoptosis. Looking at the strong association between resistance and individual PS, this approach is more appropriate with possible resistance to common PSs. In an earlier study, Park et al. studied the effect of magnetic-FeNPs in RAW264.7 cells and in a murine alveolar macrophage cell line, and showed the induction of autophagy that preceded apoptosis through mitochondrial damage and ER stress, which resulted in programmed cell death [88]. However, as interesting as this looks, care should be taken when using Fe-based nanomaterials because of their impact on normal cells, especially immune cells. 

An important compound in the body that directly scavenges the many different types of oxidant species including ROS, ^1^O_2_, superoxide anion, hydroxyl radical, hydroperoxides, peroxynitrites, lipid peroxides, nitric oxide, and carbon radicals is Glutathione [89]. Apart from the prevention of damage by cellular antioxidant defense mechanisms such as superoxide dismutases (SOD) and catalase dehydrogenases, PDT damage is also avoided when glutathione scavenges the cytotoxic products of PDT, i.e., ROS and ^1^O_2_ [90,91,92,93]. When designing PSs for PDT, therefore, in appropriate situations, certain NPs with the ability to prevent the defensive effect of glutathione should be incorporated. Ling et al. [94] studied the effect of glutathione-scavenging Poly (disulfide amide) NPs for treating cisplatin resistance cells. The study showed that the glutathione scavenging approach resulted in the reduction of glutathione and increased apoptosis of cisplatin-resistant cells. Although not much research has elucidated the usability of glutathione scavenging NPs in PDT, a similar approach seen in chemotherapy can be employed in cases of PDT resistance due to the action of glutathione. However, similar to Fe-based NPs, much consideration should be given in the design of such compounds because of their effects on normal cells. Section 4.3 below has more details.

Attenuation of resistance using compounds that directly override the mechanisms of repair and evasion of apoptosis is an important method. This shows another area where nano-based DDS is important because most of these molecules require a carrier molecule like NPs for their administration. When conjugated to NPs and PS, the three-component biomolecule has multifunctional characteristics including photosensitization, diminution of resistance, and increased stability and bioavailability. For instance, the observation that the ubiquitin-proteasome system creates a proteolytic pathway responsible for the rapid removal of PDT-damaged cellular organelles can be prevented by using proteasome inhibitors [95]. Reported by Szokalska et al. [96], the combination of proteasome inhibitors and PDT led to increased antitumor effects. Although, in the study, the cells were pretreated with the proteasome inhibiters, which is followed by PDT. These two functions can be performed simultaneously using an NP-PS-proteasome inhibiter conjugate. Similarly, in the case of resistance because of (HIF-1)-alpha in cancer cells, Broekgaarden et al. [97] noted that inhibition of the HIF-1 with acriflavine increased the efficacy of PDT by the sensitization of hypoxic tumor cells to nano-based PDT using zinc phthalocyanine-encapsulating cationic liposomes.

#### 3.1.3. Enhanced Drug Efflux

Most PS efflux mechanisms observed in PDT are those that are also associated with multi-drug efflux transporters, i.e., MDR, P-gp, and ABCG2. Cross-resistance to PDT and chemotherapy has been reported numerous times due to these ABC transporters [41,42,43,44]. To avoid PS efflux, repressing the activity of these transporters is a significant intervention. A noble example is the repression of P-gp activity by using P-gp inhibitors. Due to the important role that P-gp plays in drug efflux, a P-gp inhibitor, e.g., verapamil [98], can be considered for conjugation of a multi-functionalized NP to deliver both the photosensitizing agent and the anti-efflux drug simultaneously. Another common drug efflux molecule, ABCG2, was inhibited using tyrosine kinase inhibitors [99]. Imatinib mesylate, which is a tyrosine kinase inhibitor, increased accumulation of PS in cell lines that expressed ABCG2, and enhanced PDT efficacy both in vitro and in vivo. Many have employed the option of pretreatment with the inhibitors, which is followed by PDT. However, with the use of NPs, a simpler way through multifunctional conjugation of all molecules with NP is a potential preference.

#### 3.1.4. Resistance from Other Factors Other Than Cellular Mechanisms 

Importantly, there are other characteristics of cancer other than inherent cellular features, which confer resistance to PDT. A hypoxic tumor microenvironment is a well-established finding in most tumors. Since O_2_ is a necessary element in the PDT process, lack of O_2_ enhances tumor resistance to PDT [100]. The lack of O_2_ in itself is a limiting factor in the PDT process, and very dangerous since the presence of light in the absence of the PS substrate has a photo-bio-modulatory action, which can worsen the cancer post-PDT [11]. In certain tumors, especially deep tissue malignancies, targeting tumor hypoxia when using PDT is utterly important. Manganese dioxide (MnO_2_) NPs have a high reactivity with intracellular hydrogen peroxide (H_2_O_2_) within the tumor microenvironment to generate O_2_. These NPs have been used in several cases to enhance the efficacy of PDT in hypoxic tumors [100,101]. 

Furthermore, other NPs with additional therapeutic features, e.g., the photothermal effect of AuNp and the ability of up-convention NPs to convert low energy photons into high energy photons, are important interventions for the attenuation of resistance conferred by features of the tumor microenvironment, tumor size, and position. Recent developments in nano-based PDT aim at producing multifunctional molecules that not only possess photosensitizing features but also possess other therapeutic features that are aimed at maximizing the efficacy of PDT even in the presence of cancer resistance. A multifunctional approach is the most daring solution to the problem of PDT resistance. Zeng et al. [102] recently used a unique development that combined the photosensitizing effect with other features including:

- Catalase-like activities to decompose H_2_O_2_ to O_2_
- Glutathione consumption for enhancing PDT efficacy
- Increased PS dose
- AS1411 aptamer for nuclear targeting
- Excellent stability

## 4. Pharmacokinetic Pitfalls in Nanomedicine 

Despite being a solution for the existing cellular resistance to PDT, nano-based PDT has factors that need scrutiny in order to achieve the desired outcome. Most information regarding the use of NPs is reported from in vitro studies and, to date, the numbers of approved clinical trials and/or confirmed use of NPs in clinical practice are minimal. When in vitro research is done on NPs, the interaction between NPs, biological systems, and the immune system in vivo is somewhat overlooked. While it is true that NPs help prevent cellular resistance to PDT as described, there are a few necessary factors to consider when using NPs and their interactions with biological systems.

### 4.1. Nanoparticles and Formation of Protein Corona

**Figure 2:**
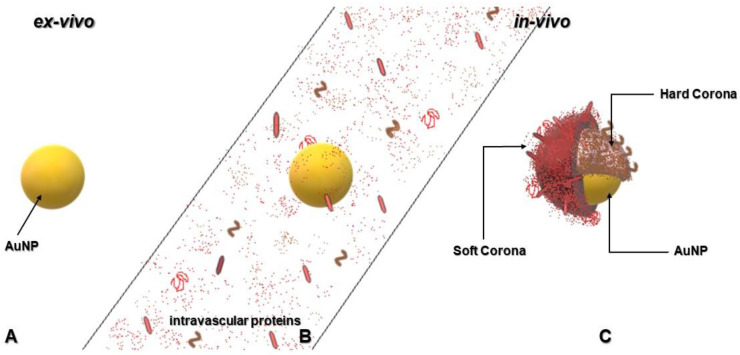
Formation of protein corona in blood plasma when NPs are injected into the blood circulation system during therapy. (**A)**. AuNPs in their original form before administration (**B**). AuNPs in the vascular system exposed to intravascular proteins (**C**). AuNPs coated with proteins and protein fragments, which renders the AuNP unrecognizable at the target site.

One characteristic of NPs that makes them useful for PDT applications is the readiness to bind other molecules by surface adsorption or formation of chemical bonds. Paradoxically, the same feature of NPs has been proven to render them inept in vivo due to the formation of protein layers around the NP, which has been termed “protein corona.” Human plasma contains a large amount of dissolved proteins [103] and, when the NP is injected intravenously into the body, these bind to the NP surface and form an adsorption layer of surrounding matter, which interferes with the NP’s physicochemical properties and defines its interactions with target cells. As shown in Figure 2 below, the formation of the protein corona alters the size, surface charge, surface composition, and functionality of NPs, which gives them a completely new biological identity [104]. Nguyen and Lee [104] described important information of protein corona emphasizing its formation, structure, and effects. Different forces including hydrogen bonding, Van der Waal interactions, electrostatic interactions, and hydrophobic interactions play a role in the bonding and/or adsorption of proteins to the NP surface [105,106]. The resulting protein corona is divided into “hard corona,” which comprises of higher affinity proteins on the NP surface that may irreversibly bind to NPs and “soft corona,” which is formed by lower affinity proteins that are reversibly bound by the NPs (Figure 2).

Nonetheless, numerous findings have presented ways of combating the effects of protein corona. Mirshafiee et al. [107] studied effects of protein pre-coating on the composition of the protein corona and the cellular uptake of NPs and demonstrated that pre-coating the surface of NPs with specific proteins to recruit similar proteins from plasma directs the formation of a protein corona enriched with predesignated plasma proteins that could be exploited for cell targeting. Therefore, this reduces the deleterious effects of the protein corona on cellular targeting and uptake. In other findings [108,109], it was shown that controlling the surface functionality of NPs modulates their physiochemistry and modify the protein corona formed on the NP surface, which, ultimately, defines its interactions with biological systems. Furthermore, the charge of NPs is very important not only for its cellular uptake, but for the type of proteins it attracts when in vivo. This phenomenon provides another means of controlling the protein corona by engineering NPs with biomolecules that inhibit interaction with proteins [110]. An effective way of increasing biocompatibility and blood circulation time is by engineering NPs with zwitterionic surfaces that will prevent the formation of protein corona. Safavi-Sohi et al. [110] used cysteine as a zwitterionic ligand and it was demonstrated that the cysteine conjugated NPs inhibited corona-induced mistargeting. Ideally, limitations in biocompatibility, cellular targeting, and blood circulation time can be overcome by engineering surface functionalized NPs with other biomolecules that prevent the influence of protein corona. A variety of coupling methods are available for conjugation. The NP surfaces are modified by conjugation to polyethylene glycol (PEG), which stabilizes the NPs by steric repulsion that inhibits colloidal aggregation in physiological conditions. Steric repulsion between individual NPs in this instance helps prevent aggregation of the NPS by enabling the NPs to repel one another. This occurs due to either one or the combination of the osmotic effect (i.e., high concentration of PEG chains in the region of overlap) and the volume restriction effect.

### 4.2. Nanoparticles and the Immune System

It is somewhat ironic that the unique properties these molecules have in the nanoscale dimensions can be beneficial to biological systems and harmful to health, at times. The immune system in a healthy individual is designed to recognize and effect upon any foreign material for elimination. Metal-based NPs such as Au, may be toxic and stimulate an immune inflammatory reaction, and activate the complement system in vivo [111,112]. Poland et al. [113] studied the effect of the administering carbon nanotubes in mice and reported the induction of inflammation and the formation of granulomatous lesions. Nanomaterials, especially metal NPs, are recognized as foreign by the immune system but, incidentally, if the immune system categorizes them to be unharmful, they are ignored or tolerated [114]. Hence, the design and physicochemical properties of NPs such as size, shape, surface charge, and solubility in water are extremely important to their interaction with the immune system and NPs can be designed to either inhibit the immune system, enhance it, or simply avoid recognition [115]. 

On the other hand, certain metal NPs, especially FeNPs, discussed in Section 3.1.2 above, have a lethal effect on the immune system [116]. These NPs are toxic to cells of the immune system and suppress the function of human T lymphocytes. Therefore, although they are suggested as a potential compound in the attenuation of resistance due to modulation of autophagy, their use in PDT needs to be assessed with more focus given to the option of using them in DDS to direct them to the targeted cancerous tissues using monoclonal antibodies (mAbs) or other targeting molecules. Lower doses are also an important factor to consider in order to minimize toxicity of immune cells. 

For drug delivery, designing NPs to escape immune recognition is very important. The available interventions that prevent immune recognition are also effective against the formation of protein corona, as described previously. Polyethylene glycol (PEG) is one molecule that has good resistance against nonspecific adsorption and, in many studies, has been used in the engineering of multifunctional NPs for drug delivery [80,106,117]. Additionally, when NPs are coated with PEG, they are shielded from opsonization, aggregation, and phagocytosis and the systemic circulation time is prolonged [117]. Idyllically, by means of hybrid nanostructured carriers using copolymers, polypeptoids and multi-functionalized DDS, the hitches in the applications of nanomaterials for PS delivery can be eliminated [118,119,120]. Ultimately, the development of a DDS that employs the use of nanomaterials should require a detailed assessment of the preferred therapeutic outcome, the mode of administration, the characteristics of the target tissue, and its microenvironment.

### 4.3. Nanoparticles and Their Toxicity to Healthy Tissue

Another adverse effect of certain NPs, which is not pleasant to hear, seeing the many advantages they have, is their significant toxicity to normal tissue. Apart from their effect on immune cells, the oxidative stress produced by Fe-based NPs in neural tissue can cause side effects including neural degeneration [121]. The effect of these NPs have also been linked to certain neurodegenerative conditions including Parkinson’s and Alzheimer’s diseases [121,122,123]. Therefore, using Fe-based NPs should be done with proper assessment and dose-management. Additionally, with reference to the choice of NPs when treating patients, these NPs can be avoided in patients with underlying neurologic conditions or those at risk of neurodegenerative diseases. ZnO-NPs have also been studied in PDT of certain cancers [124,125]. However, many reports of these NPs include their high ROS induction rate, which leads to cell death through autophagic vacuole accumulation and mitochondria damage in normal skin cells [126]. These observations require the need to carefully consider the dose, type of cancer, and underlying patient conditions. 

Section 3.1 above described the important role that Poly (disulfide amide) NPs play in scavenging glutathione in cancer cells. However, it is very important to know that glutathione is a very important molecule in the body. Because of its important role and the high concentration in most healthy cells, glutathione is often referred to as the “master” antioxidant and is responsible for removing oxidants in healthy cells. In line with this, it is necessary to manage the proper use and administration of glutathione scavenging NPs. This is another example where targeted delivery of the PDT compound is important. The design of PDT agents comprising these NPs should include cancer cell-targeting molecules to avoid their accumulation in normal tissue. 

## 5. Conclusions

In summary, present studies have adequately demonstrated cellular resistance to PDT in many cancer cell lines. In this case, we examined the mechanisms of PDT and the acquisition of resistance to PDT. The resistance mechanisms that have been reported are complex and PS-specific, which range from PS efflux to intrinsic cellular signaling after treatment. We, therefore, conclude that altering the photosensitizing molecule using nanotechnology is an ideal paradigm for the enhancement of PDT efficacy in the presence of cellular resistance. Most importantly, because of the nature of resistance, a multifunctional approach using modified PSs, nanomaterials, cell targeting ligands, mAbs, and other biomolecules to produce a single photosensitizing compound with other intrinsic features, is the most recommended direction to take in the advancement of PDT.

Prospective research perspectives should include studies on the co-delivery of therapeutic agents to a target site in one constructed biomolecule other than mere combination therapy by administering two or more therapies individually. This will minimize risk of treatment failure due to resistance since most resistance patterns, unlike efflux, are conferred to individual drugs or PS. This is possible by using multi-functionalized carrier molecules such as PEGylated NP-PS-Chemo conjugates, PEGylated NP-PS-Aptamer conjugates, PEGylated NP-PS-mAb conjugates, and PEGylated NP-PS1-PS2-mAb/Aptamer conjugates (i.e., conjugating two or more PSs in one biomolecule). However, this should be done with proper scrutiny and taking into account that the final photophysical and physicochemical properties of the molecule are conducive for biological systems and relevant for the therapeutic action. Researchers should also take advantage of novel elements that show photoactive properties in other applications. A good example is the cage structure carbon nanomaterial, C_60_ fullerene, which has been studied for its unique photophysical and photochemical properties since the 1980s. A PS-C_60_–PEG should be studied for its potential use in attenuating some of the discussed cellular mechanisms.

Additionally, although a considerable amount of biomarkers to specific tumors have been recognized along the years, research still need to be conducted in search for new targets in cancers including gene modulation, elements of the tumor microenvironment, and cellular response pathways, which would all aid in the betterment of novel drug designs and combinatory therapies.

## Figures and Tables

**Table 1:** Photodynamic therapy (PDT) resistance mechanisms.

Proposed Mechanism(s)	Cell Line	Photosensitizer(s)	Reference
MDR Mediated drug efflux *	P388/ADR murine leukemia	Copper benzochlorin iminium salt, a cationic PS	[41]
ABCG2 associated drug efflux *	NCI-H1650 MX50 bronchoalveolar carcinoma	Pyropheophorbide	[42,43,44]
		Chlorin e6	
		PpIX from 5-aminolevulinic acid (ALA)	
Endocytic vesicle localization of TPPS2a	MES-SA/Dx5 cells	Disulfonated meso-tetraphenylporphine (TPPS2a)	[45]
Modulation of the PS uptake and/or subcellular localization as well as changes in mitochondrial size and function	RIF-1 fibrosarcoma	Photofrin II	[46,47,48]
		Polyhematoporphyrin (PHP)	
		Zinc (II) pyridinium-substituted phthalocyanine (ZnPCP)	
Alterations in the enzymes of the heme pathway that produces PpIX	murine mammary adenocarcinoma	5-aminolevulinic acid (ALA)	[49]
Attenuation of light in tissue ^LD^	human glioma spheroids	5-aminolevulinic acid (ALA)	[50]
Delayed apoptotic response ^sp^	P388 murine leukemia	tin octaethylpurpurin amidine (SnOPA)	[51]

* Also associated with chemotherapy resistance, ^sp^ signaling pathways, ^LD^ light-dose dependent resistance.

**Table 2:** Nanomaterials used in oncology.

Nanomaterial	Description	Application	Reference(s)
Nanoparticles (NPs)	NPs are nanosized colloidal particles with a polymeric matrix that can adsorb or bind a therapeutic compound. NPs can be classified as metallic NPs, polymeric NPs (PNPs) and solid lipid NPs (SLNs), depending on the material of which they are made.	Chemotherapy PDT	[61]
Quantum dots	Semiconductor particles with an inert polymer coating. The material used for the core can be chosen depending on the emission wavelength range being targeted. Targeted molecules can be attached to the coating.	Cancer imaging PDT	[62,63]
Carbon nanotubes	Cylinder-like assemblies of carbon atoms with cross-sectional dimensions in the nanometer range, and lengths that can extend over a thousand times their diameters.	Biomarker detection chemotherapy	[64,65]
Dendrimers	These polymers possess an architecture that gives them an alterable size and shape with several branches around an inner core.	PDT Chemotherapy Cancer imaging	[66,67,68]
Liposomes	Uni/multilamellar nanosized carrier molecules made of lipids surrounding a water core, formed from the dispersion of phospholipids in an aqueous medium.	PDT Chemotherapy	[69,70]
Nanowires and nanocantilever arrays	Nanocantilever are flexible beams that can be coated with molecules capable of binding to cancer biomarkers. When certain biomolecular interactions occur on one surface of a microcantilever beam, the cantilever bends and can be detected. Nanoscale sensing wires that can be coated with molecules such as antibodies to bind to proteins of interest and transmit their information through electrodes to computers.	Biomarker detection Early detection of precancerous and malignant lesions from biological fluids	[71]
Liquid Crystalline Systems	Also called anisotropic phase, they are polymers that lie between the boundaries of solid substances and liquids when in melt state, and, macroscopically, in the melt state, they are fluids.	Transdermal delivery of vitamins	[72]
